# Wnt signaling networks in autism spectrum disorder and intellectual disability

**DOI:** 10.1186/s11689-016-9176-3

**Published:** 2016-12-05

**Authors:** Vickie Kwan, Brianna K. Unda, Karun K. Singh

**Affiliations:** Department of Biochemistry and Biomedical Sciences, Stem Cell and Cancer Research Institute, McMaster University, Hamilton, Ontario L8S 4K1 Canada

**Keywords:** Autism spectrum disorders, ASD, Synapse, Wnt signaling, GSK3, Neurodevelopment, Signaling, Plasticity, Mutations, Neurotransmission, Neurogenesis, Neuronal migration

## Abstract

**Background:**

Genetic factors play a major role in the risk for neurodevelopmental disorders such as autism spectrum disorders (ASDs) and intellectual disability (ID). The underlying genetic factors have become better understood in recent years due to advancements in next generation sequencing. These studies have uncovered a vast number of genes that are impacted by different types of mutations (e.g., de novo, missense, truncation, copy number variations).

**Abstract:**

Given the large volume of genetic data, analyzing each gene on its own is not a feasible approach and will take years to complete, let alone attempt to use the information to develop novel therapeutics. To make sense of independent genomic data, one approach is to determine whether multiple risk genes function in common signaling pathways that identify signaling “hubs” where risk genes converge. This approach has led to multiple pathways being implicated, such as synaptic signaling, chromatin remodeling, alternative splicing, and protein translation, among many others. In this review, we analyze recent and historical evidence indicating that multiple risk genes, including genes denoted as high-confidence and likely causal, are part of the Wingless (Wnt signaling) pathway. In the brain, Wnt signaling is an evolutionarily conserved pathway that plays an instrumental role in developing neural circuits and adult brain function.

**Conclusions:**

We will also review evidence that pharmacological therapies and genetic mouse models further identify abnormal Wnt signaling, particularly at the synapse, as being disrupted in ASDs and contributing to disease pathology.

## Background

### The emerging genetic landscape of Wnt signaling in ASDs

ASDs and other psychiatric disorders may have heritability estimates greater than 90% [1], suggesting a strong genetic component to disease. With this background in mind, there has been an enormous advancement of new genetic technologies to discover risk-causing genes and loci. These developments paired with an increased ability to process large data sets have led to many new risk genes being discovered. The number of genes and chromosomal loci linked to ASDs is growing, making it difficult to determine which one(s) to study. This has inspired the field to determine if there are links between the genes and whether they converge into signaling networks important for proper brain development. While the spectrum of ASDs is reflected by the multiple individual risk genes and loci, there is some common denominator between affected individuals, which strongly suggests that disruption of the core neurodevelopmental signaling pathways leads to disease symptoms. In this review, we will examine accumulating evidence for the involvement of Wnt signaling in developmental cognitive disorders. This includes emerging genetic data from large sequencing studies, clinically used medications, and mouse models. We will also present potential avenues for therapeutic approaches, and how Wnt signaling may be modulated for treatment of patient symptoms by leveraging clinical trial data from other fields.

### Making sense of genetic findings

It is no surprise that the clinical heterogeneity of ASDs can be explained, at least in part, by the large number of genetic mutations found through next-generation sequencing. The number of mutations discovered ranges in the several hundreds, according to well-known sources such as the Simons Foundation Autism Research Initiative (SFARI). There is much difficulty in trying to understand the biological etiology of ASDs when so many genes are involved. One hypothesis is that the genetic lesions disrupt specific signaling pathways during discrete time points of brain development. For example, many of the initial genetic studies identified genes involved in synapse development and refinement. This was largely based on findings that multiple genes in syndromic forms of ASDs (e.g., Fragile X syndrome (*FMR1*), Rett syndrome (*MECP2*), Angelman syndrome (*UBE3A*), and genes that cause non-syndromic forms of ASDs (e.g., the Shank and neuroligin/neurexin family of proteins) have important roles during synapse development and refinement [2–8]. This suggests that the disruption of postnatal synaptic maturation could increase the risk for developing ASDs and related disorders. However, there has been accumulating evidence that other brain developmental milestones are also vulnerable, such as prenatal brain development (e.g., neurogenesis) [9], or postnatal development of non-neuronal cells (e.g., oligodendrocytes during myelination and microglia function) [10–13]. This is also supported by the implication of discrete cell types in the brain based on the identification of specific risk genes expressed in those cells (e.g., inhibitory neurons) [14–16]. In the current review, we take an alternative approach that is not in contrast to these hypotheses but examines whether there is convergence of multiple risk genes onto specific signaling pathways, which ultimately impact multiple cell types and/or developmental processes. We put forth the notion that by focusing on a specific pathway and dissecting which molecular players in that pathway are important for disease pathophysiology, it may offer an opportunity to identify key proteins to be pharmacologically targeted by drug therapies to treat these disorders.

## Review

### Convergent evidence for Wnt signaling

**Fig. 1:**
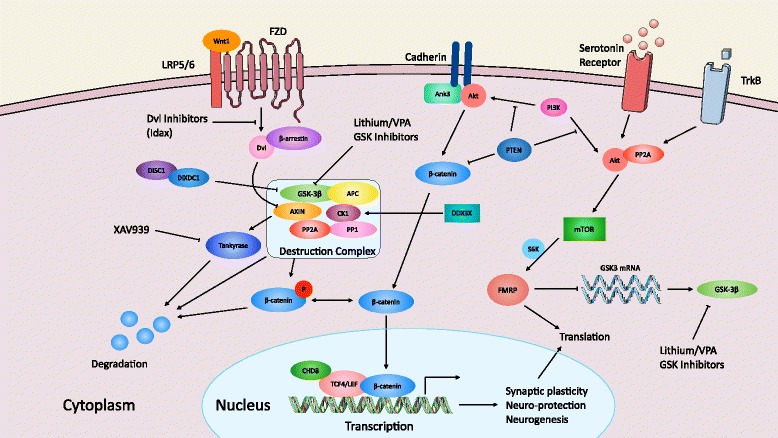
Summary of genetic and pharmacological evidence implicating Wnt signaling in developmental cognitive disorders. This diagram depicts the canonical Wnt signaling pathway which consists of Wnt binding to the Frizzled-LRP5/6 co-receptor complex and inhibiting the disassembly of the destruction complex which results in stabilized β-catenin levels, translocation to the nucleus, and initiation of Wnt-dependent gene transcription. Several genes in the canonical Wnt pathway are also identified as high-risk genes associated with autism and intellectual disability (*CHD8*, *DDX3X*, and *TCF4*). Furthermore, listed are a number of pharmacological agents and drugs that target Wnt signaling molecules and can modulate the pathway. Many of the genes listed are shared between different upstream ligand-receptor pathways

One pathway highlighted in the multitude of genetic studies is the Wingless (Wnt) signaling. This pathway is highly studied and conserved from lower to higher organisms, where it plays a variety of roles in almost all tissues. Broadly speaking, Wnt signaling in the brain can be divided into two main pathways: (i) “canonical” signaling that results in the stabilization of the protein β-catenin (encoded by *CTNNB1*), which upon stabilization, can exert functions at the plasma membrane or in the nucleus and can act as a transcription factor that modulates the expression of target genes (Fig. 1); and (ii) “non-canonical” β-catenin-independent signaling [17]. Interestingly, many of the proteins in both signaling pathways localize to the synapse and play important functions in synaptic growth and maturation [18–23]. There are now multiple lines of evidence implicating this pathway in the etiology and pathophysiology of ASD and intellectual disability (ID). While the human genetic data is an important supporting factor, it is not the only one. There are a number of mouse genetic knockout (KO) models targeting Wnt signaling molecules, describing molecular, cellular, electrophysiological, and behavioral deficits that are consistent with ASD and ID. Furthermore, the genes involved in Wnt signaling are of significant clinical interest because there are a variety of approved drugs that either inhibit or stimulate this pathway.

### Genetic evidence implicating Wnt signaling genes and support by cellular models

#### CHD8

The strongest single candidate gene for non-syndromic ASDs is chromodomain helicase DNA binding protein 8 (CHD8) [24–30]. There are multiple de novo, truncating, or missense mutations discovered in *CHD8* in individuals with ASDs [27–29, 31–34]. CHD8 is found at active transcription sites with histone modifications H3K4me3 or H3K27ac, and it is thought to directly activate genes by binding near the transcriptional start site and promoting transcription factor activity or recruitment. It can also indirectly impact transcription by interacting with modified histone sites and other co-regulators to make chromatin more assessable [24, 34–36]. Interestingly, one of the major pathways regulated by CHD8 is canonical Wnt signaling [37, 38]. Previous work characterized CHD8 as a negative regulator of canonical Wnt signaling, which fits with the hypothesis that elevated canonical Wnt signaling activity causes excessive proliferation of embryonic neural progenitor cells (NPCs) in the brain and may in part explain the macrocephaly (“big brain”) phenotype observed in patients [27]. Furthermore, recent studies in human neural progenitors lacking one copy of *CHD8* support this notion, as it revealed many target genes controlled by CHD8 that are involved in the regulation of spine head size [34, 39, 40]. However a recent study discovered that CHD8 is in fact a positive regulator of Wnt/β-catenin signaling NPCs, while simultaneously demonstrating that it negatively regulates the pathway in non-neuronal cell lines [41]. Given this unexpected finding, this suggests that CHD8 regulates Wnt signaling in a cell-specific manner, and the possibility that some of the *CHD8* mutations may not be as simple as loss-of-function for Wnt signaling. Further work is needed to clarify how CHD8 regulates Wnt signaling in different cell types in the brain, and how patients with *CHD8* mutations acquire macrocephaly. It is also important to note that Wnt signaling is only one neurodevelopmental pathway regulated by CHD8, and recent studies have identified many others (e.g., chromatin remodeling). Therefore, future work needs to determine the precise mechanisms and time points during which CHD8 regulates Wnt signaling during neurodevelopment. This is important to better comprehend how prenatal brain development could be compromised in individuals with *CHD8* mutations.

#### CTNNB1 (β-catenin)

β-catenin is a central player in the canonical Wnt signaling pathway and works with co-factors to initiate Wnt-dependent gene transcription (Fig. 1). It has directly been implicated in ASDs due to the identification of de novo mutations in the *CTNNB1* gene in patients with ASD using exome sequencing [25, 28, 29, 42]. Given the core nature of this gene in canonical Wnt signaling, this strongly places aberrations in Wnt signaling as one of the main networks in ASD pathogenesis. Network analysis from gene expression data also indicates that β-catenin exists in a protein network, including CHD8 and other ASD or ID associated genes [25]. The relationship between Wnt signaling and chromatin remodeling factors demonstrates that proper interplay between these pathways is important for appropriate levels of canonical Wnt-dependent gene transcription. CHD8 regulates β-catenin-mediated canonical Wnt signaling, which could occur by CHD8 directly by binding to β-catenin or indirectly by inhibiting the recruitment of co-factors required for transcription at promoter sequences. Future work will need to determine precisely which neural progenitor sub-populations are most sensitive to disruptions in canonical Wnt/β-catenin signaling during prenatal brain development. For example, previous studies indicated that global stabilization of β-catenin in the cortex, which elevates canonical Wnt signaling, leads to brain overgrowth due to increased cycling neural progenitor cells and production of neurons [43]. In contrast, deletion of β-catenin from parvalbumin-expressing inhibitory neurons leads to ASD-like defects in neuronal activation and behavior, such as social interaction and object recognition impairments [44].

β-caxtenin also functions in pathways other than canonical Wnt signaling, such as cell adhesion at the plasma membrane. β-catenin interacts with cadherins to regulate dendritic spine growth and synaptic competition during the process of postnatal period of dendritic pruning [45]. A recent study identified that dominant mutations in * CTNNB1 *from ID patients when expressed in mice have a reduced affinity for membrane-associated cadherins [46]. This is associated with a decrease in cadherin interaction and decreased intrahemispheric connections, with deficits in dendritic branching, long-term potentiation, and cognitive function [46]. Therefore, it is possible that mutations in β-catenin could lead to aberrant Wnt signaling developmentally, concurrent with disruption of plasma membrane signaling at synaptic sites, both leading to disrupted gene transcription programs and abnormal synaptic plasticity. Furthermore, both of these pathogenic events would increase disease risk. In this regard, these pathways are also likely connected given that β-catenin signaling at the membrane impacts nuclear Wnt-dependent transcription [47].

#### PTEN

Another high-risk autism candidate gene that has roles in Wnt signaling is *PTEN* due to the discovery of numerous individuals with mutations [29, 48–50]. PTEN has multiple functions but is best known for its role as a negative regulator of the PI3K-Akt-mTOR pathway. Individuals with heterozygous mutations in * PTEN* are also at risk for macrocephaly, indicating that PTEN regulates brain size, which is thought to be an impact on certain individuals with ASD [51–59]. Multiple mouse models of *Pten* have further strengthened the notion that it is an important regulator of different neural circuits associated with ASDs and cognition. For example, an early study identified that global *Pten* +/- mice have impairments in social interaction behaviors [52]. Knocking out *Pten* in the cerebellar Purkinje cells led to impaired sociability, repetitive behavior, and deficits in motor learning. Additionally, knocking out *Pten* in a subset of cortical excitatory neurons results in profound synaptic signaling changes [60], while ASD-associated *Pten* alleles expressed in inhibitory neurons cause excitatory/inhibitory imbalances [54]. Interestingly, PTEN has recently been identified to function with β-catenin to regulate normal brain growth, implicating PTEN in Wnt signaling. It was discovered that β-catenin signaling is elevated in a mouse model of *Pten* (*Pten* +/-), and a heterozygous mutation in *β-catenin* suppresses the excessive cortical brain growth in *Pten* *+/-* mice [53]. This indicates that multiple ASD risk factors likely converge upon neural progenitor proliferation during embryonic brain development, potentially through regulation of canonical Wnt signaling.

#### TCF7L2 (TCF4)

Recent studies have identified mutations in *CTNNB1* within individuals with developmental delay and ID who do not have an ASD [46, 61, 62]. Additional support comes from another ASD exome sequencing study that identified de novo loss of function variants in *TCF7L2 *(transcription factor 7-like 2 (T-cell specific, HMG-box)) [63, 64], which also goes by the name *TCF4*, and is confused with transcription factor 4 (TCF4) as they share the same symbol. Importantly, TCF7L2 is a key player in canonical Wnt signaling as it helps to initiate gene transcriptional response when Wnt ligands bind their receptors on the membrane, and the signal is transduced to the nucleus (Fig. 1). These genetic studies directly implicate de novo missense variants in *TCF4* in ASD, suggesting that perturbations to the core canonical signaling complex play a pathogenic role. However, the role of TCF4 in brain development and which time points and cell types it regulates is not well known and needs to be identified in future studies.

#### DDX3X

A recent study identified de novo mutations in *DEAD-box helicase 3* *, X-linked *(*DDX3X*) in a population of unexplained ID [65]. DDX3X was identified as a regulator of the Wnt-β-catenin network, via regulation of the kinase activity of CK1ε, to promote phosphorylation of Dvl, both critical factors in canonical Wnt signaling (Fig. 1). Moreover, in model systems testing the Wnt pathway, DDX3X was found to be required for Wnt-β-catenin signaling in mammalian cells through loss of function studies [66]. Therefore, in addition to ASD, Wnt-β-catenin signaling may be disrupted in generalized ID and developmental disorders (DD), further demonstrating the importance of this pathway in proper neurodevelopment. However, a critical question that remains is how do mutations in Wnt signaling molecules give rise to different phenotypes, such as ASD versus ID/DD? This insight would provide tremendous clinical utility, as it will allow caregivers and clinicians to plan therapies according to the future outcome.

### Animal models support the involvement of Wnt signaling

Mouse models provide support for Wnt signaling as a clinically relevant pathway for developmental cognitive disorders. First, modeling of high-risk (i.e., causative) ASD genes, for example using gene knockouts (KO), offer the opportunity to determine the neural circuits and brain regions responsible for causing ASD-like behavior. Second, interrogation of other genes in the same pathway, which are not directly involved in ASD from human genetic studies, offers the opportunity to further support that pathway in ASD pathophysiology. For example, while no ASD-specific genetic mutations have been identified in the *disheveled* genes (*Dvl 1, 2 and 3*), *Dvl1* or *Dvl1/3 *KO mice display adult social and repetitive behavioral abnormalities, which are the core features of ASD symptoms [67–69]. This type of example lends further evidence that perturbation of the core Wnt signaling transduction molecules like Dvl1/3 can result in ASD-like abnormalities even though they are not directly implicated in human genetic studies.

In addition to Dvl1/3, recent studies have highlighted that conditional or complete KO mouse models of other genes involved in Wnt signaling support the pathway being involved in ASD-like phenotypes. One of the best-studied genes is *glycogen synthase kinase 3 *(*GSK3*) α and β, which is a negative regulator of canonical Wnt signaling and also plays important roles directly at the synapse (Fig. 1). It is well-established that the inhibition of GSK3 using lithium or specific inhibitors (e.g., CHIR 99021) causes an increase in activation of the canonical transcriptional pathway of Wnt signaling [70]. *Gsk-3β* heterozygous (+/-) mice display behaviors that resemble wild type (WT) mice treated with lithium, a drug that is used to treat bipolar disorder [71], demonstrating that disruption of Wnt signaling leads to behavioral abnormalities. Furthermore, forebrain-specific deletion of *Gsk-3β* in excitatory neurons leads to anxiolytic and pro-social effects [72], suggesting that GSK-3β plays important roles in normal behavior. The most convincing evidence that GSK3 and Wnt signaling may be involved in developmental cognitive disorders is its role in Fragile X syndrome (FXS), which is the most commonly inherited form of intellectual disability and is linked to ASDs [73, 74]. Fragile X Mental Retardation Protein (FMRP) KO mice, which is a FXS model, has been shown to possess a dysregulation of GSK3β activity. Specifically, GSK-3β protein and its activity is pathogenically elevated in FXS models [75, 76], and pharmacological correction of this enhanced activity using lithium or GSK3 inhibitors in mice rescues neurobehavioral and brain morphological abnormalities [77–82]. Furthermore, studies investigating FXS mice demonstrated that Wnt signaling is also disrupted [83, 84]. Of course, GSK-3β has many downstream signaling targets, one of which is the Wnt signaling pathway; demonstrating that modulation of GSK-3β activity can have therapeutic effects beyond the treatment of bipolar disorder.

Another well-studied gene in relation to ASD and psychiatric disorders is *disrupted in schizophrenia 1* (*DISC1*). While the genetic evidence linking DISC1 to developmental cognitive disorders is not strong, the multiple cellular and mouse models of *Disc1* perturbation has led to important findings linking Wnt signaling to abnormal neurodevelopment. For example, a landmark study initially described DISC1 as an inhibitor of GSK-3β, similar to the actions of lithium, demonstrating that DISC1 positively regulates canonical Wnt signaling [85], which has been followed up by other studies [86–89]. Several follow-up studies on multiple mouse models of *Disc1* demonstrate that DISC1 perturbation causes significant neurodevelopmental phenotypes, including cognitive defects and psychiatric-like behavioral manifestations [90–94].

There are other known regulators of Wnt signaling that when disrupted leads to neurocognitive and neurodevelopmental phenotypes. A recent example is *AnkyrinG *(*Ank3*), which was found to possess a genome-wide significant signal in bipolar disorder [95, 96]. Ank3 is a scaffolding protein that localizes to the nodes of Ranvier in mature neurons, important for the formation and maintenance of the axon initial segment [95]. It has also been shown to regulate glutamatergic synapse structure and function through modulation of AMPAR-mediated synaptic transmission and maintenance of dendritic spine morphology [97]. Interestingly, Ank3 is a negative regulator of canonical Wnt signaling during embryonic neurogenesis in the mouse brain and functionally interacts with DISC1 to regulate this process [46]. *Ank3* heterozygous mice possess behavioral phenotypes such as reduced anxiety and increased motivation for reward, which can be corrected by modulating Wnt signaling through GSK-3β [98], demonstrating the clinical involvement of this pathway. In addition to *Ank3*, other recently characterized genes in mice also support a role for Wnt signaling in neurodevelopmental disorders. DIX domain containing 1 (DIXDC1) is a positive regulator of Wnt signaling and neurogenesis through binding to DISC1 [99] and a *Dixdc1* KO mouse displayed behaviors associated with neuropsychiatric disorders such as abnormal startle reflex and reduced social interaction [100]. The behavioral phenotypes displayed by these mice could be rescued through lithium or GSK3 inhibitor treatment [101, 102].

There are three other Wnt signaling-related genes that have been characterized in mice that lend further support to the involvement of Wnt signaling in developmental cognitive disorders (see Fig. 1). The first is *adenomatous polyposis coli* (*APC*), which is a critical component of the destruction complex in the canonical Wnt pathway and is important for neural plasticity, learning, and memory in mice. A conditional *Apc* KO mouse showed increased synaptic spine density, elevated frequency of miniature excitatory postsynaptic potentials (mEPSPs), enhanced long-term potentiation (LTP), and ASD-like behaviors (e.g., repetitive behaviors and reduced social interest) [103]. Second, analysis of a *Prickle2 *mouse model demonstrates its importance in ASD-related neural circuits and behavior [104, 105]. Prickle2 is a postsynaptic protein that interacts with PSD-95 and is part of the non-canonical Wnt signaling pathway [106]. The *Prickle2 *KO mouse has previously been shown to be more sensitive to seizures and also shows reduced dendrite branching, synapse number, and postsynaptic density (PSD) size, as well as behavioral abnormalities (learning abnormalities, altered social interaction, and behavioral inflexibility). Although the involvement of Prickle2 implicates non-canonical Wnt signaling, the phenotypes associated with the KO mouse demonstrate that multiple aspects of Wnt signaling (canonical and non-canonical) are important for the establishment of neural circuits that are disrupted in ASD. While the studies on APC and Prickle2 do not directly implicate abnormal Wnt signaling, we speculate that these mice would have alterations in this pathway due to the importance of these molecules in Wnt signaling in neural cells. Third, a recent study identified that rare missense variants in the *Wnt1* gene discovered in ASD patients show abnormal activation of the Wnt signaling pathway, providing evidence that subtle changes to the coding sequence of Wnt signaling molecules alter biological signaling [107]. Together, these studies indicate that when analyzed using animal models, members of the Wnt signaling pathway, which have no link to disease from human genetic studies, demonstrate how disruption of this core signaling pathway in the brain results in developmental phenotypes consistent with human disease.

### Targeting Wnt signaling in ASD/ID mouse models

In addition to animal models, there are two specific drug-induced models that implicate Wnt signaling. The first is valproic acid (VPA), which is thought to increase the risk for ASD through exposure to a pregnant woman during prenatal development [108]. The administration of VPA to pregnant mice has long been used as a model of ASD, as the offspring of these mice develop ASD-like deficits in brain structure, neuronal signaling, and behavior [109]. VPA has many targets, but one of its better characterized effects is the stimulation of the canonical Wnt signaling pathway through modulation of histone deacetylase and GSK3 [110–114], demonstrating that abnormal Wnt signaling likely plays an important role in the pathogenicity of VPA. A second model that was recently developed and more specifically implicates Wnt signaling is exposure of pregnant mice to the compound, XAV939, which is a tankyrase inhibitor, resulting in enhanced canonical Wnt signaling [115]. This leads to the expansion of the intermediate progenitor cell population in the developing cerebral cortex. The result of exposure to XAV939 is an overpopulation of neurons in the cortex, which disrupts the development and function of dendrites and dendritic spines of excitatory neurons and alters the distribution of interneurons. These mice exhibit ASD-like behavioral abnormalities, implicating that changes to canonical Wnt signaling during prenatal brain development can have a profound impact on brain size and function. These results suggest a causal relationship between abnormal modulation of Wnt signaling during neurodevelopment and autism-like features [115].

### Hope for ASD treatment using Wnt signaling modulators?

There is only one FDA approved for ASDs, which is used to treat irritability associated with ASDs (risperidone, an antipsychotic medication), demonstrating the urgent need to find new medications. Many of the medications used are “off-label” (e.g., antidepressants, anticonvulsants, stimulants, and antianxiety medications) and do not treat the core symptoms, and can have very strong side effects. While these medications have multiple modes of molecular action, interestingly, many impact Wnt signaling. For example, haloperidol (typical antipsychotic medication) is known to inhibit dopamine receptors, thereby increasing GSK3β inhibition through Akt activation [116], which impacts downstream canonical Wnt signaling [117, 118]. Selective serotonin reuptake inhibitors (SSRIs) (e.g., fluoxetine), which are used to treat depression, potentially by increasing hippocampal neurogenesis in mice, have been shown to antagonize canonical Wnt signaling, which causes a reduction in expression of the serotonin transporter (SERT) in serotonergic raphe neurons through miR-16 [119, 120]. Additionally, lithium is a well-known treatment for bipolar disorder, and one of its main activities is inhibition of GSK-3β, which positively stimulates the canonical Wnt pathway [121–123]. Stimulants such as Methylphenidate (e.g., Ritalin) can function as a negative regulator of the canonical pathway by activating GSK-3β [124, 125]. In this regard, various GSK-3β inhibitors have been used to rescue neurogenesis defects in mouse models of psychiatric disorders and ASD, which also stimulate canonical Wnt signaling pathways [69]. Taken together, while it is important to be cautious of the multiple mechanisms of action of all of the classes of medications discussed here, it is intriguing to find that all of them either directly or indirectly impact canonical Wnt signaling in the brain to some degree. This suggests that abnormal Wnt signaling likely plays a core role in the disease pathogenesis of developmental cognitive disorders, and restoring normal levels of this pathway with medications could be an option for treatment.

Many times the medications tested in pilot clinical trials for neurological disorders are failed drugs from cancer trials. There are many drugs developed and tested as modulators of Wnt signaling in the cancer field that could potentially be repurposed for developmental cognitive disorders. In cases where a reduction in Wnt signaling is thought to underlie the pathology of the disorder, usage of compounds that elevated canonical Wnt signaling could be applied. An example of this is GSK-3β inhibitors that have failed in cancer trials but may be effective for ASDs and ID (e.g., Tideglusig, ClinicalTrials.gov identifier: NCT02586935). In cases where elevated Wnt signaling is thought to contribute to disease pathology, there are many potential options to inhibit canonical Wnt signaling using chemicals (Fig. 1) that inhibit the interaction between β-catenin and its targets (e.g., inhibiting β-catenin interaction with the TCF factors), disheveled inhibitors (through targeting of the PDZ domain which generally inhibit the Frizzled–PDZ interaction), and tankyrase inhibitors (e.g., XAV939, which induces the stabilization of axin by inhibiting the poly (ADP)-ribosylating enzymes tankyrase 1 and tankyrase 2) [126]. These candidate compounds may be of clinical use in cases where it is thought that the genetic risk factor for ASD or ID causes elevated canonical Wnt signaling (e.g., potentially some individuals with CHD8 mutations); however, even if these drugs made it to the clinic, they would likely have to be delivered in utero*,* since embryonic brain development is most affected by such genetic mutations, posing ethical issues for pre-diagnosis therapies.

## Conclusions

The goal towards better understanding and treating developmental cognitive disorders is a difficult road and will require a multifaceted research and clinical approach to be successful. In this review, we present evidence that one such signaling pathway that may be central to disease pathogenesis and treatment is the Wnt signaling network. Ongoing and future genetic studies will need to determine the strength of association of this pathway with disease; however, given the medications and drugs that target this pathway currently available, this presents an opportunity for new clinical trials in the near future.

## Abbreviations

ANK3: AnkyrinG
APC: Adenomatous polyposis coli
ASD: Autism spectrum disorder
BP: Bipolar disorder
CHD8: Chromatin-helicase-DNA-binding protein 8
CIHR: Chiron
CTNNB1: (β-catenin)
DDX3X: DEAD-box helicase 3, X-linked
DISC1: Disrupted in schizophrenia 1
DIXDC1: Dix domain containing 1
DVL: Disheveled
FDA: Food and drug administration
FMR1: Fragile X mental retardation 1
FMRP: Fragile X mental retardation protein
FXS: Fragile X syndrome
GSK: Glycogen synthase kinase
ID: Intellectual disability
KO: Knockout
LTP: Long-term potentiation
MECP2: Methyl CpG binding protein 2; miniature excitatory postsynaptic potentials
mEPSPs: Miniature excitatory postsynaptic potentials
NDD: Neurodevelopmental disorders
Prickle2: Prickle planar cell polarity protein 2
PSD: Postsynaptic density
PTEN: Phosphatase and tensin homolog
SERT: Serotonin transporter
SFARI: Simons Foundation Autism Research Initiative
SSRI: Selective serotonin reuptake inhibitor
UBE3A: Ubiquitin-protein ligase E3A
VPA: Valproic acid
Wnt: Wingless
WT: Wild type

## References

[1] Lichtenstein P, Carlstrom E, Rastam M, Gillberg C, Anckarsater H (2010). The genetics of autism spectrum disorders and related neuropsychiatric disorders in childhood. Am J Psychiatry.

[2] Ebrahimi-Fakhari D, Sahin M (2015). Autism and the synapse: emerging mechanisms and mechanism-based therapies. Curr Opin Neurol.

[3] Shepherd GM, Katz DM (2011). Synaptic microcircuit dysfunction in genetic models of neurodevelopmental disorders: focus on Mecp2 and Met. Curr Opin Neurobiol.

[4] Mullins C, Fishell G, Tsien RW (2016). Unifying views of autism spectrum disorders: a consideration of autoregulatory feedback loops. Neuron.

[5] Habela CW, Song H, Ming GL (2016). Modeling synaptogenesis in schizophrenia and autism using human iPSC derived neurons. Mol Cell Neurosci.

[6] Huber KM, Klann E, Costa-Mattioli M, Zukin RS (2015). Dysregulation of mammalian target of rapamycin signaling in mouse models of autism. J Neurosci.

[7] Bourgeron T (2015). From the genetic architecture to synaptic plasticity in autism spectrum disorder. Nat Rev Neurosci.

[8] Volk L, Chiu SL, Sharma K, Huganir RL (2015). Glutamate synapses in human cognitive disorders. Annu Rev Neurosci.

[9] Packer A (2016). Neocortical neurogenesis and the etiology of autism spectrum disorder. Neurosci Biobehav Rev.

[10] Pacey LK, Xuan IC, Guan S, Sussman D, Henkelman RM, Chen Y, Thomsen C, Hampson DR (2013). Delayed myelination in a mouse model of fragile X syndrome. Hum Mol Genet.

[11] Ameis SH, Catani M (2015). Altered white matter connectivity as a neural substrate for social impairment in Autism Spectrum Disorder. Cortex.

[12] Estes ML, McAllister AK (2015). Immune mediators in the brain and peripheral tissues in autism spectrum disorder. Nat Rev Neurosci.

[13] Bilimoria PM, Stevens B (2015). Microglia function during brain development: new insights from animal models. Brain Res.

[14] Gao R, Penzes P (2015). Common mechanisms of excitatory and inhibitory imbalance in schizophrenia and autism spectrum disorders. Curr Mol Med.

[15] Marin O (2012). Interneuron dysfunction in psychiatric disorders. Nat Rev Neurosci.

[16] Nelson SB, Valakh V (2015). Excitatory/inhibitory balance and circuit homeostasis in autism spectrum disorders. Neuron.

[17] Salinas PC, Zou Y (2008). Wnt signaling in neural circuit assembly. Annu Rev Neurosci.

[18] Caracci MO, Avila ME, De Ferrari GV (2016). Synaptic Wnt/GSK3beta signaling hub in autism. Neural Plast.

[19] Purro SA, Galli S, Salinas PC (2014). Dysfunction of Wnt signaling and synaptic disassembly in neurodegenerative diseases. J Mol Cell Biol.

[20] Oliva CA, Vargas JY, Inestrosa NC (2013). Wnts in adult brain: from synaptic plasticity to cognitive deficiencies. Front Cell Neurosci.

[21] Stamatakou E, Salinas PC (2014). Postsynaptic assembly: a role for Wnt signaling. Dev Neurobiol.

[22] Budnik V, Salinas PC (2011). Wnt signaling during synaptic development and plasticity. Curr Opin Neurobiol.

[23] Okerlund ND, Cheyette BN (2011). Synaptic Wnt signaling—a contributor to major psychiatric disorders?. J Neurodev Disord.

[24] Barnard RA, Pomaville MB, O’Roak BJ (2015). Mutations and modeling of the chromatin remodeler CHD8 define an emerging autism etiology. Front Neurosci.

[25] Krumm N, O’Roak BJ, Shendure J, Eichler EE (2014). A de novo convergence of autism genetics and molecular neuroscience. Trends Neurosci.

[26] Sanders SJ (2015). First glimpses of the neurobiology of autism spectrum disorder. Curr Opin Genet Dev.

[27] Bernier R, Golzio C, Xiong B, Stessman HA, Coe BP, Penn O, Witherspoon K, Gerdts J, Baker C, Vulto-van Silfhout AT (2014). Disruptive CHD8 mutations define a subtype of autism early in development. Cell.

[28] O’Roak BJ, Vives L, Girirajan S, Karakoc E, Krumm N, Coe BP, Levy R, Ko A, Lee C, Smith JD (2012). Sporadic autism exomes reveal a highly interconnected protein network of de novo mutations. Nature.

[29] O’Roak BJ, Vives L, Fu W, Egertson JD, Stanaway IB, Phelps IG, Carvill G, Kumar A, Lee C, Ankenman K (2012). Multiplex targeted sequencing identifies recurrently mutated genes in autism spectrum disorders. Science.

[30] Krumm N, Turner TN, Baker C, Vives L, Mohajeri K, Witherspoon K, Raja A, Coe BP, Stessman HA, He ZX (2015). Excess of rare, inherited truncating mutations in autism. Nat Genet.

[31] Neale BM, Kou Y, Liu L, Ma’ayan A, Samocha KE, Sabo A, Lin CF, Stevens C, Wang LS, Makarov V (2012). Patterns and rates of exonic de novo mutations in autism spectrum disorders. Nature.

[32] Talkowski ME, Rosenfeld JA, Blumenthal I, Pillalamarri V, Chiang C, Heilbut A, Ernst C, Hanscom C, Rossin E, Lindgren AM (2012). Sequencing chromosomal abnormalities reveals neurodevelopmental loci that confer risk across diagnostic boundaries. Cell.

[33] McCarthy SE, Gillis J, Kramer M, Lihm J, Yoon S, Berstein Y, Mistry M, Pavlidis P, Solomon R, Ghiban E (2014). De novo mutations in schizophrenia implicate chromatin remodeling and support a genetic overlap with autism and intellectual disability. Mol Psychiatry.

[34] Sugathan A, Biagioli M, Golzio C, Erdin S, Blumenthal I, Manavalan P, Ragavendran A, Brand H, Lucente D, Miles J (2014). CHD8 regulates neurodevelopmental pathways associated with autism spectrum disorder in neural progenitors. Proc Natl Acad Sci U S A.

[35] Wilkinson B, Grepo N, Thompson BL, Kim J, Wang K, Evgrafov OV, Lu W, Knowles JA, Campbell DB (2015). The autism-associated gene chromodomain helicase DNA-binding protein 8 (CHD8) regulates noncoding RNAs and autism-related genes. Transl Psychiatry.

[36] Cotney J, Muhle RA, Sanders SJ, Liu L, Willsey AJ, Niu W, Liu W, Klei L, Lei J, Yin J (2015). The autism-associated chromatin modifier CHD8 regulates other autism risk genes during human neurodevelopment. Nat Commun.

[37] Thompson BA, Tremblay V, Lin G, Bochar DA (2008). CHD8 is an ATP-dependent chromatin remodeling factor that regulates beta-catenin target genes. Mol Cell Biol.

[38] Nishiyama M, Skoultchi AI, Nakayama KI (2012). Histone H1 recruitment by CHD8 is essential for suppression of the Wnt-β-catenin signaling pathway. Mol Cell Biol.

[39] Wang P, Lin M, Pedrosa E, Hrabovsky A, Zhang Z, Guo W, Lachman HM, Zheng D (2015). CRISPR/Cas9-mediated heterozygous knockout of the autism gene CHD8 and characterization of its transcriptional networks in neurodevelopment. Mol Autism.

[40] Merner N, Forgeot d’Arc B, Bell SC, Maussion G, Peng H, Gauthier J, Crapper L, Hamdan FF, Michaud JL, Mottron L (2016). A de novo frameshift mutation in chromodomain helicase DNA-binding domain 8 (CHD8): a case report and literature review. Am J Med Genet A.

[41] Durak O, Gao F, Kaeser-Woo YJ, Rueda R, Martorell AJ, Nott A, Liu CY, Watson LA, Tsai LH. Chd8 mediates cortical neurogenesis via transcriptional regulation of cell cycle and Wnt signaling. Nat Neurosci. 2016. 10.1038/nn.4400 PMC5386887 27694995

[42] Sanders SJ, Murtha MT, Gupta AR, Murdoch JD, Raubeson MJ, Willsey AJ, Ercan-Sencicek AG, DiLullo NM, Parikshak NN, Stein JL (2012). De novo mutations revealed by whole-exome sequencing are strongly associated with autism. Nature.

[43] Chenn A, Walsh CA (2002). Regulation of cerebral cortical size by control of cell cycle exit in neural precursors. Science.

[44] Dong F, Jiang J, McSweeney C, Zou D, Liu L, Mao Y. Deletion of CTNNB1 in inhibitory circuitry contributes to autism-associated behavioral defects. Hum Mol Genet. 2016. Epub ahead of print. 10.1093/hmg/ddw131 PMC5181638 27131348

[45] Bian WJ, Miao WY, He SJ, Qiu Z, Yu X (2015). Coordinated spine pruning and maturation mediated by inter-spine competition for cadherin/catenin complexes. Cell.

[46] Tucci V, Kleefstra T, Hardy A, Heise I, Maggi S, Willemsen MH, Hilton H, Esapa C, Simon M, Buenavista MT (2014). Dominant beta-catenin mutations cause intellectual disability with recognizable syndromic features. J Clin Invest.

[47] Zhang J, Shemezis JR, McQuinn ER, Wang J, Sverdlov M, Chenn A (2013). AKT activation by N-cadherin regulates beta-catenin signaling and neuronal differentiation during cortical development. Neural Dev.

[48] Spinelli L, Black FM, Berg JN, Eickholt BJ, Leslie NR (2015). Functionally distinct groups of inherited PTEN mutations in autism and tumour syndromes. J Med Genet.

[49] Frazier TW, Embacher R, Tilot AK, Koenig K, Mester J, Eng C (2015). Molecular and phenotypic abnormalities in individuals with germline heterozygous PTEN mutations and autism. Mol Psychiatry.

[50] McBride KL, Varga EA, Pastore MT, Prior TW, Manickam K, Atkin JF, Herman GE (2010). Confirmation study of PTEN mutations among individuals with autism or developmental delays/mental retardation and macrocephaly. Autism Res.

[51] Page DT, Kuti OJ, Prestia C, Sur M (2009). Haploinsufficiency for Pten and Serotonin transporter cooperatively influences brain size and social behavior. Proc Natl Acad Sci U S A.

[52] Kwon CH, Luikart BW, Powell CM, Zhou J, Matheny SA, Zhang W, Li Y, Baker SJ, Parada LF (2006). Pten regulates neuronal arborization and social interaction in mice. Neuron.

[53] Chen Y, Huang WC, Sejourne J, Clipperton-Allen AE, Page DT (2015). Pten mutations alter brain growth trajectory and allocation of cell types through elevated beta-catenin signaling. J Neurosci.

[54] Vogt D, Cho KK, Lee AT, Sohal VS, Rubenstein JL (2015). The parvalbumin/somatostatin ratio is increased in Pten mutant mice and by human PTEN ASD alleles. Cell Rep.

[55] Clipperton-Allen AE, Page DT (2015). Decreased aggression and increased repetitive behavior in Pten haploinsufficient mice. Genes Brain Behav.

[56] Clipperton-Allen AE, Page DT (2014). Pten haploinsufficient mice show broad brain overgrowth but selective impairments in autism-relevant behavioral tests. Hum Mol Genet.

[57] Takeuchi K, Gertner MJ, Zhou J, Parada LF, Bennett MV, Zukin RS (2013). Dysregulation of synaptic plasticity precedes appearance of morphological defects in a Pten conditional knockout mouse model of autism. Proc Natl Acad Sci U S A.

[58] Tilot AK, Frazier TW, Eng C (2015). Balancing proliferation and connectivity in PTEN-associated autism spectrum disorder. Neurotherapeutics.

[59] Zhou J, Parada LF (2012). PTEN signaling in autism spectrum disorders. Curr Opin Neurobiol.

[60] Lugo JN, Smith GD, Arbuckle EP, White J, Holley AJ, Floruta CM, Ahmed N, Gomez MC, Okonkwo O (2014). Deletion of PTEN produces autism-like behavioral deficits and alterations in synaptic proteins. Front Mol Neurosci.

[61] Dubruc E, Putoux A, Labalme A, Rougeot C, Sanlaville D, Edery P (2014). A new intellectual disability syndrome caused by CTNNB1 haploinsufficiency. Am J Med Genet A.

[62] Kuechler A, Willemsen MH, Albrecht B, Bacino CA, Bartholomew DW, van Bokhoven H, van den Boogaard MJ, Bramswig N, Buttner C, Cremer K (2015). De novo mutations in beta-catenin (CTNNB1) appear to be a frequent cause of intellectual disability: expanding the mutational and clinical spectrum. Hum Genet.

[63] Iossifov I, O’Roak BJ, Sanders SJ, Ronemus M, Krumm N, Levy D, Stessman HA, Witherspoon KT, Vives L, Patterson KE (2014). The contribution of de novo coding mutations to autism spectrum disorder. Nature.

[64] De Rubeis S, He X, Goldberg AP, Poultney CS, Samocha K, Cicek AE, Kou Y, Liu L, Fromer M, Walker S (2014). Synaptic, transcriptional and chromatin genes disrupted in autism. Nature.

[65] Snijders Blok L, Madsen E, Juusola J, Gilissen C, Baralle D, Reijnders MR, Venselaar H, Helsmoortel C, Cho MT, Hoischen A (2015). Mutations in DDX3X are a common cause of unexplained intellectual disability with gender-specific effects on Wnt signaling. Am J Hum Genet.

[66] Cruciat CM, Dolde C, de Groot RE, Ohkawara B, Reinhard C, Korswagen HC, Niehrs C (2013). RNA helicase DDX3 is a regulatory subunit of casein kinase 1 in Wnt-β-catenin signaling. Science.

[67] Lijam N, Paylor R, McDonald MP, Crawley JN, Deng CX, Herrup K, Stevens KE, Maccaferri G, McBain CJ, Sussman DJ (1997). Social interaction and sensorimotor gating abnormalities in mice lacking Dvl1. Cell.

[68] Long JM, LaPorte P, Paylor R, Wynshaw-Boris A (2004). Expanded characterization of the social interaction abnormalities in mice lacking Dvl1. Genes Brain Behav.

[69] Belinson H, Nakatani J, Babineau BA, Birnbaum RY, Ellegood J, Bershteyn M, McEvilly RJ, Long JM, Willert K, Klein OD (2016). Prenatal beta-catenin/Brn2/Tbr2 transcriptional cascade regulates adult social and stereotypic behaviors. Mol Psychiatry.

[70] Meijer L, Flajolet M, Greengard P (2004). Pharmacological inhibitors of glycogen synthase kinase 3. Trends Pharmacol Sci.

[71] O’Brien WT, Harper AD, Jove F, Woodgett JR, Maretto S, Piccolo S, Klein PS (2004). Glycogen synthase kinase-3β haploinsufficiency mimics the behavioral and molecular effects of lithium. J Neurosci.

[72] Latapy C, Rioux V, Guitton MJ, Beaulieu JM (2012). Selective deletion of forebrain glycogen synthase kinase 3β reveals a central role in serotonin-sensitive anxiety and social behaviour. Philos Trans R Soc Lond B Biol Sci.

[73] Bhakar AL, Dolen G, Bear MF (2012). The pathophysiology of fragile X (and what it teaches us about synapses). Annu Rev Neurosci.

[74] Bear MF, Huber KM, Warren ST (2004). The mGluR theory of fragile X mental retardation. Trends Neurosci.

[75] Comery TA, Harris JB, Willems PJ, Oostra BA, Irwin SA, Weiler IJ, Greenough WT (1997). Abnormal dendritic spines in fragile X knockout mice: maturation and pruning deficits. Proc Natl Acad Sci U S A.

[76] Mines MA, Yuskaitis CJ, King MK, Beurel E, Jope RS (2010). GSK3 influences social preference and anxiety-related behaviors during social interaction in a mouse model of fragile X syndrome and autism. PLoS One.

[77] Franklin AV, King MK, Palomo V, Martinez A, McMahon LL, Jope RS (2014). Glycogen synthase kinase-3 inhibitors reverse deficits in long-term potentiation and cognition in fragile X mice. Biol Psychiatry.

[78] Mines MA, Jope RS (2011). Glycogen synthase kinase-3: a promising therapeutic target for fragile x syndrome. Front Mol Neurosci.

[79] Yuskaitis CJ, Mines MA, King MK, Sweatt JD, Miller CA, Jope RS (2010). Lithium ameliorates altered glycogen synthase kinase-3 and behavior in a mouse model of fragile X syndrome. Biochem Pharmacol.

[80] Min WW, Yuskaitis CJ, Yan Q, Sikorski C, Chen S, Jope RS, Bauchwitz RP (2009). Elevated glycogen synthase kinase-3 activity in Fragile X mice: key metabolic regulator with evidence for treatment potential. Neuropharmacology.

[81] Chen X, Sun W, Pan Y, Yang Q, Cao K, Zhang J, Zhang Y, Chen M, Chen F, Huang Y (2013). Lithium ameliorates open-field and elevated plus maze behaviors, and brain phospho-glycogen synthase kinase 3-beta expression in fragile X syndrome model mice. Neurosciences.

[82] Guo W, Murthy AC, Zhang L, Johnson EB, Schaller EG, Allan AM, Zhao X (2012). Inhibition of GSK3β improves hippocampus-dependent learning and rescues neurogenesis in a mouse model of fragile X syndrome. Hum Mol Genet.

[83] Matic K, Eninger T, Bardoni B, Davidovic L, Macek B (2014). Quantitative phosphoproteomics of murine Fmr1-KO cell lines provides new insights into FMRP-dependent signal transduction mechanisms. J Proteome Res.

[84] Luo Y, Shan G, Guo W, Smrt RD, Johnson EB, Li X, Pfeiffer RL, Szulwach KE, Duan R, Barkho BZ (2010). Fragile x mental retardation protein regulates proliferation and differentiation of adult neural stem/progenitor cells. PLoS Genet.

[85] Mao Y, Ge X, Frank CL, Madison JM, Koehler AN, Doud MK, Tassa C, Berry EM, Soda T, Singh KK (2009). Disrupted in schizophrenia 1 regulates neuronal progenitor proliferation via modulation of GSK3beta/beta-catenin signaling. Cell.

[86] Singh KK, Ge X, Mao Y, Drane L, Meletis K, Samuels BA, Tsai LH (2010). Dixdc1 is a critical regulator of DISC1 and embryonic cortical development. Neuron.

[87] Boccitto M, Doshi S, Newton IP, Nathke I, Neve R, Dong F, Mao Y, Zhai J, Zhang L, Kalb R (2016). Opposing actions of the synapse-associated protein of 97-kDa molecular weight (SAP97) and Disrupted in Schizophrenia 1 (DISC1) on Wnt/β-catenin signaling. Neuroscience.

[88] Srikanth P, Han K, Callahan DG, Makovkina E, Muratore CR, Lalli MA, Zhou H, Boyd JD, Kosik KS, Selkoe DJ (2015). Genomic DISC1disruption in hiPSCs alters Wnt signaling and neural cell fate. Cell Rep.

[89] Singh KK, De Rienzo G, Drane L, Mao Y, Flood Z, Madison J, Ferreira M, Bergen S, King C, Sklar P (2011). Common DISC1 polymorphisms disrupt Wnt/GSK3beta signaling and brain development. Neuron.

[90] Seshadri S, Faust T, Ishizuka K, Delevich K, Chung Y, Kim SH, Cowles M, Niwa M, Jaaro-Peled H, Tomoda T (2015). Interneuronal DISC1 regulates NRG1-ErbB4 signalling and excitatory-inhibitory synapse formation in the mature cortex. Nat Commun.

[91] Clapcote SJ, Lipina TV, Millar JK, Mackie S, Christie S, Ogawa F, Lerch JP, Trimble K, Uchiyama M, Sakuraba Y (2007). Behavioral phenotypes of Disc1 missense mutations in mice. Neuron.

[92] Abazyan B, Nomura J, Kannan G, Ishizuka K, Tamashiro KL, Nucifora F, Pogorelov V, Ladenheim B, Yang C, Krasnova IN (2010). Prenatal interaction of mutant DISC1 and immune activation produces adult psychopathology. Biol Psychiatry.

[93] Furukubo-Tokunaga K, Kurita K, Honjo K, Pandey H, Ando T, Takayama K, Arai Y, Mochizuki H, Ando M, Kamiya A, et al. DISC1 causes associative memory and neurodevelopmental defects in fruit flies. Mol Psychiatry. 2016. 10.1038/mp.2016.15 PMC4993648 26976042

[94] Saito A, Taniguchi Y, Rannals MD, Merfeld EB, Ballinger MD, Koga M, Ohtani Y, Gurley DA, Sedlak TW, Cross A, et al. Early postnatal GABA receptor modulation reverses deficits in neuronal maturation in a conditional neurodevelopmental mouse model of DISC1. Mol Psychiatry. 2016. 10.1038/mp.2015.203 PMC4935661 26728564

[95] Durak O, de Anda FC, Singh KK, Leussis MP, Petryshen TL, Sklar P, Tsai LH (2015). Ankyrin-G regulates neurogenesis and Wnt signaling by altering the subcellular localization of β-catenin. Mol Psychiatry.

[96] Iqbal Z, Vandeweyer G, van der Voet M, Waryah AM, Zahoor MY, Besseling JA, Roca LT, Vulto-van Silfhout AT, Nijhof B, Kramer JM (2013). Homozygous and heterozygous disruptions of ANK3: at the crossroads of neurodevelopmental and psychiatric disorders. Hum Mol Genet.

[97] Smith KR, Kopeikina KJ, Fawcett-Patel JM, Leaderbrand K, Gao R, Schurmann B, Myczek K, Radulovic J, Swanson GT, Penzes P (2014). Psychiatric risk factor ANK3/ankyrin-G nanodomains regulate the structure and function of glutamatergic synapses. Neuron.

[98] Leussis MP, Berry-Scott EM, Saito M, Jhuang H, de Haan G, Alkan O, Luce CJ, Madison JM, Sklar P, Serre T (2013). The ANK3 bipolar disorder gene regulates psychiatric-related behaviors that are modulated by lithium and stress. Biol Psychiatry.

[99] Singh KK. Dixdc1 is a critical regulator of DISC1 and embryonic cortical development supplemental information. Neuron. 2010. 10.1016/j.neuron.2010.06.002 PMC2938013 20624590

[100] Kivimae S, Martin PM, Kapfhamer D, Ruan Y, Heberlein U, Rubenstein JL, Cheyette BN (2011). Abnormal behavior in mice mutant for the Disc1 binding partner, Dixdc1. Transl Psychiatry.

[101] Martin PM, Stanley RE, Ross AP, Freitas AE, Moyer CE, Brumback AC, Iafrati J, Stapornwongkul KS, Dominguez S, Kivimae S, et al. DIXDC1 contributes to psychiatric susceptibility by regulating dendritic spine and glutamatergic synapse density via GSK3 and Wnt/beta-catenin signaling. Mol Psychiatry. 2016. Epub ahead of print. 10.1038/mp.2016.184 PMC5395363 27752079

[102] Kwan V, Meka DP, White SH, Hung CL, Holzapfel NT, Walker S, Murtaza N, Unda BK, Schwanke B, Yuen RK, Habing K, Milsom C, Hope KJ, Truant R, Scherer SW, Calderon de Anda F, Singh KK. DIXDC1 Phosphorylation and Control of Dendritic Morphology Are Impaired by Rare Genetic Variants. Cell Rep. 2016 Nov 8;17(7):1892-1904. doi: 10.1016/j.celrep.2016.10.047. 10.1016/j.celrep.2016.10.047 27829159

[103] Zhou XL, Giacobini M, Anderlid BM, Anckarsater H, Omrani D, Gillberg C, Nordenskjold M, Lindblom A (2007). Association of adenomatous polyposis coli (APC) gene polymorphisms with autism spectrum disorder (ASD). Am J Med Genet B Neuropsychiatr Genet.

[104] Sowers LP, Loo L, Wu Y, Campbell E, Ulrich JD, Wu S, Paemka L, Wassink T, Meyer K, Bing X (2013). Disruption of the non-canonical Wnt gene PRICKLE2 leads to autism-like behaviors with evidence for hippocampal synaptic dysfunction. Mol Psychiatry.

[105] Sowers LP, Mouw TJ, Ferguson PJ, Wemmie JA, Mohapatra DP, Bassuk AG (2013). The non-canonical Wnt ligand Wnt5a rescues morphological deficits in Prickle2-deficient hippocampal neurons. Mol Psychiatry.

[106] Nagaoka T, Tabuchi K, Kishi M (2015). PDZ interaction of Vangl2 links PSD-95 and Prickle2 but plays only a limited role in the synaptic localisation of Vangl2. Scientific reports.

[107] Martin PM, Yang X, Robin N, Lam E, Rabinowitz JS, Erdman CA, Quinn J, Weiss LA, Hamilton SP, Kwok PY (2013). A rare WNT1 missense variant overrepresented in ASD leads to increased Wnt signal pathway activation. Transl Psychiatry.

[108] Christensen J, Gronborg TK, Sorensen MJ, Schendel D, Parner ET, Pedersen LH, Vestergaard M (2013). Prenatal valproate exposure and risk of autism spectrum disorders and childhood autism. Jama.

[109] Hall AC, Brennan A, Goold RG, Cleverley K, Lucas FR, Gordon-Weeks PR, Salinas PC (2002). Valproate regulates GSK-3-mediated axonal remodeling and synapsin I clustering in developing neurons. Mol Cell Neurosci.

[110] Wang L, Liu Y, Li S, Long ZY, Wu YM (2015). Wnt signaling pathway participates in valproic acid-induced neuronal differentiation of neural stem cells. Int J Clin Exp Pathol.

[111] Wiltse J (2005). Mode of action: inhibition of histone deacetylase, altering WNT-dependent gene expression, and regulation of beta-catenin—developmental effects of valproic acid. Crit Rev Toxicol.

[112] Zhang Y, Sun Y, Wang F, Wang Z, Peng Y, Li R (2012). Downregulating the canonical Wnt/β-catenin signaling pathway attenuates the susceptibility to autism-like phenotypes by decreasing oxidative stress. Neurochem Res.

[113] Go HS, Kim KC, Choi CS, Jeon SJ, Kwon KJ, Han SH, Lee J, Cheong JH, Ryu JH, Kim CH (2012). Prenatal exposure to valproic acid increases the neural progenitor cell pool and induces macrocephaly in rat brain via a mechanism involving the GSK-3β/β-catenin pathway. Neuropharmacology.

[114] Phiel CJ, Zhang F, Huang EY, Guenther MG, Lazar MA, Klein PS (2001). Histone deacetylase is a direct target of valproic acid, a potent anticonvulsant, mood stabilizer, and teratogen. J Biol Chem.

[115] Fang WQ, Chen WW, Jiang L, Liu K, Yung WH, Fu AK, Ip NY (2014). Overproduction of upper-layer neurons in the neocortex leads to autism-like features in mice. Cell Rep.

[116] Emamian ES, Hall D, Birnbaum MJ, Karayiorgou M, Gogos JA (2004). Convergent evidence for impaired AKT1-GSK3beta signaling in schizophrenia. Nat Genet.

[117] Sutton LP, Honardoust D, Mouyal J, Rajakumar N, Rushlow WJ (2007). Activation of the canonical Wnt pathway by the antipsychotics haloperidol and clozapine involves dishevelled-3. J Neurochem.

[118] Sutton LP, Rushlow WJ (2011). The effects of neuropsychiatric drugs on glycogen synthase kinase-3 signaling. Neuroscience.

[119] Launay JM, Mouillet-Richard S, Baudry A, Pietri M, Kellermann O (2011). Raphe-mediated signals control the hippocampal response to SRI antidepressants via miR-16. Transl Psychiatry.

[120] Baudry A, Mouillet-Richard S, Schneider B, Launay JM, Kellermann O (2010). miR-16 targets the serotonin transporter: a new facet for adaptive responses to antidepressants. Science.

[121] Klein PS, Melton DA (1996). A molecular mechanism for the effect of lithium on development. Proc Natl Acad Sci U S A.

[122] Hedgepeth CM, Conrad LJ, Zhang J, Huang HC, Lee VM, Klein PS (1997). Activation of the Wnt signaling pathway: a molecular mechanism for lithium action. Dev Biol.

[123] Zhang F, Phiel CJ, Spece L, Gurvich N, Klein PS (2003). Inhibitory phosphorylation of glycogen synthase kinase-3 (GSK-3) in response to lithium. Evidence for autoregulation of GSK-3. J Biol Chem.

[124] Mines MA, Jope RS (2012). Brain region differences in regulation of Akt and GSK3 by chronic stimulant administration in mice. Cell Signal.

[125] Mines MA, Beurel E, Jope RS (2013). Examination of methylphenidate-mediated behavior regulation by glycogen synthase kinase-3 in mice. Eur J Pharmacol.

[126] Kahn M (2014). Can we safely target the WNT pathway?. Nat Rev Drug Discov.

